# Localized Surface Hydrophilicity Tailoring of Polyimide Film for Flexible Electronics Manufacturing Using an Atmospheric Pressure Ar/H_2_O Microplasma Jet

**DOI:** 10.3390/mi13111853

**Published:** 2022-10-29

**Authors:** Bowen Ji, Tao Wang, Meng Li, Liping Shi, Xiaoli You, Fanqi Sun, Haiwen Luan

**Affiliations:** 1 Unmanned System Research Institute, Northwestern Polytechnical University, Xi’an 710072, China; 2 Collaborative Innovation Center, Northwestern Polytechnical University, Shanghai 201108, China; 3 School of Mechanical Engineering, Anhui University of Technology, Ma’anshan 243032, China; 4 Querrey Simpson Institute for Bioelectronics, Northwestern University, Evanston, IL 60201, USA

**Keywords:** microplasma jet, polyimide film micromachining, surface modification, hydrophilicity tailoring

## Abstract

The poor hydrophilicity of polyimide (PI) films limits their applications in flexible electronics, such as in wearable and implantable bio-MEMS devices. In this paper, an atmospheric pressure Ar/H_2_O microplasma jet (μAPPJ) with a nozzle diameter of 100 μm was utilized to site-selectively tune the surface hydrophilicity of a PI film. The electrical and optical characteristics of the μAPPJ were firstly investigated, and the results showed that multi-spikes occurred during the plasma discharge and that diverse reactive species, such as O atoms and OH radicals, were generated in the plasma plume. The physical and chemical properties of pristine and microplasma-modified PI surfaces were characterized by the water contact angle (WCA), atomic force microscopy (AFM) and X-ray photoelectron spectroscopy (XPS). The wettability of the PI surface was significantly enhanced after microplasma modification, and the WCA could be adjusted by varying the applied voltage, water vapor content, plasma treatment time and storage time. The AFM images indicated that the surface roughness increased after the plasma treatment, which partially contributed to an improvement in the surface hydrophilicity. The XPS results showed a reduction in the C content and an increase in the O content, and abundant hydrophilic polar oxygen-containing functional groups were also grafted onto the PI film surface. Finally, the interaction mechanism between the PI molecular chains and the microplasma is discussed. The breaking of C-N and C-O bonds and the grafting of OH radicals were the key pathways to dominate the reaction process.

## 1. Introduction

Recently, flexible electronics such as wearable and implantable devices, flexible batteries and flexible supercapacitors have attracted tremendous attention in the fields of health monitoring, human–machine interactions, energy storage, etc. [1,2,3,4,5]. Differently from traditional rigid devices, flexible electronics mainly utilize flexible polymer substrates to replace traditional silicon or glass to ensure that the devices can maintain the advantages of flexibility, stretchability, and excellent biocompatibility [6]. As promising polymer substrates, polyimide (PI) films have been extensively used in diverse flexible devices due to their excellent thermal stability, mechanical properties, chemical resistance, and low dielectric constant [7,8,9,10]. However, the nature of inertness and the low surface energy of PI films makes it difficult for strong bonding to occur between PI and other materials, such as metal [11]. Therefore, it is of great significance to modify PI surfaces to obtain the desired surface properties without changing their bulk properties.

Diverse methods have been proposed to modify PI surfaces, such as chemical processing, photo-irradiation, and plasma treatments [12,13,14]. Among them, plasma treatment is considered the most effective and eco-friendly method due to the advantages of its dry process, low cost, easy operation and rich of various active species. Up to now, different kinds of plasma processing technologies have been reported for PI modification, including low-pressure plasma and atmospheric-pressure plasma treatment [15,16,17]. Low-pressure plasma treatment makes the surface characteristics of the modified films relatively uniform. However, due to the need for expensive vacuum equipment, a cumbersome operation, and difficulty with selectively controlling the modified area, it is greatly limited in the field of PI film surface modification. In contrast, atmospheric-pressure plasma can directly treat the film surface without the usage of vacuum equipment, which greatly reduces the cost and time of film surface processing. By ejecting the plasma from the generation region to form a plasma jet, direct contact between the processed objects and the discharge region can be avoided, thus greatly expanding the application range of atmospheric-pressure plasmas [18]. However, most of the atmospheric pressure plasma jets reported so far for PI film surface treatment have used plasma jets with diameters ranging from several millimeters to several centimeters, which makes it difficult to accurately control the processing area of the film surfaces. Actually, in most cases, it is not necessary to modify the entire surface of a flexible substrate; it is only necessary to perform local processing on target regions [19,20]. Therefore, it is necessary to develop a new atmospheric pressure microplasma jet to meet the requirements for the localized processing of polyimide films.

In this paper, an atmospheric pressure Ar/H_2_O microplasma jet (μAPPJ) with a diameter of about 100 μm was proposed and used for the localized surface modification of a polyimide film. The electrical and optical characteristics of the μAPPJ and the effect of different applied voltages, water vapor contents and plasma treatment times on the hydrophilicity of PI films were investigated and discussed. This work could provide a promising method for the in situ and localized surface hydrophilicity tuning of PI films for flexible electronics.

## 2. Experiment Details

### 2.1. Microplasma Jet Setup

**Figure 1:**
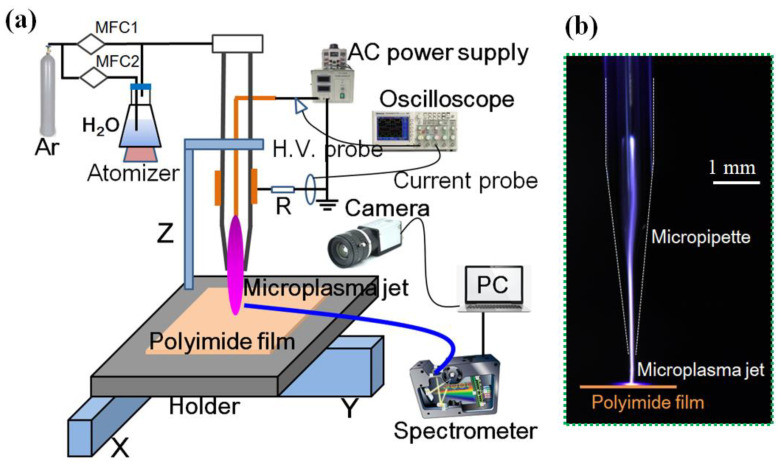
(**a**) Schematic diagram of the experimental setup for localized surface modification of PI film using μAPPJ; (**b**) photograph of the generated microplasma jet.

The experimental setup of the atmospheric pressure Ar/H_2_O microplasma jet is presented in Figure 1. The plasma generator was composed of a quartz micropipette with an inner diameter of 0.5 mm and a nozzle diameter of 100 μm, respectively. A needle–ring electrode structure was used in this experiment. A stainless-steel wire with a diameter of 100 μm was used as the needle electrode and directly inserted into the micropipette with its tip 4 mm away from the tube nozzle. A copper foil with a thickness of 150 μm and a width of 5 mm was utilized as the ring electrode and wrapped around the tube with its end 7 mm away from the tube exit. A sinusoidal AC high-voltage source (CTP-2000, Nanjing Suman Co., Ltd., Nanjing, China) was used as the power supply, and its high-voltage port was connected to the needle electrode while its ground port was connected to the ring electrode. An argon (Ar) and water (H_2_O) vapor mixture was introduced into the micropipette through a bubbling bottle with distilled water, and the H_2_O vapor content was adjusted by the auxiliary Ar flow. The two gas flows were respectively controlled by two mass flowmeters, and the total gas flow was kept at 400 sccm in this experiment. The water vapor content in the gas mixture was calculated through the flow ratio between Ar passing through the water and the total gas flow [21]. A PI film was directly placed on the acrylic holder of an XY platform and the μAPPJ was vertically installed on the PI sample, with the working distance fixed at 1 mm.

### 2.2. Diagnostic Methods

The discharge images of the μAPPJ were captured with a digital camera. The electrical characteristics of the microplasma jets were detected with a high-voltage probe (P6015A) and a current probe (Pearson 2100), and the data were recorded by an oscilloscope (Tektronix TBS1102, USA). The optical emission spectroscopy (OES) of the microplasma jet was collected with a spectrometer (MAX2000-Pro Spectrometer, Shanghai Wyoptics Co., Ltd., Shanghai, China) with a variable wavelength from 200 nm to 1100 nm. The hydrophilicity of the PI film surfaces was evaluated using water contact angle (WCA) measurements. All the WCAs were measured using the sessile drop method with a contact angle meter (SDC-350, Sindin Co., Ltd., Guangzhou, China) at room temperature. The morphologies of the PI film surfaces were observed with an atomic force microscope (AFM, FM-Nanoview1000, Feishiman Co., Ltd., Suzhou, China) in the non-contact mode with a detection region of 4 × 4 μm^2^. The chemical elements of the PI film surfaces were analyzed with an X-ray photoelectron spectrometer (XPS, AXIS ULTRA DLD, Kratos, USA) with an excitation source of Al Kα radiation (λ = 1486.6 eV).

## 3. Results and Discussion

### 3.1. Electrical Characteristics of μAPPJ

**Figure 2:**
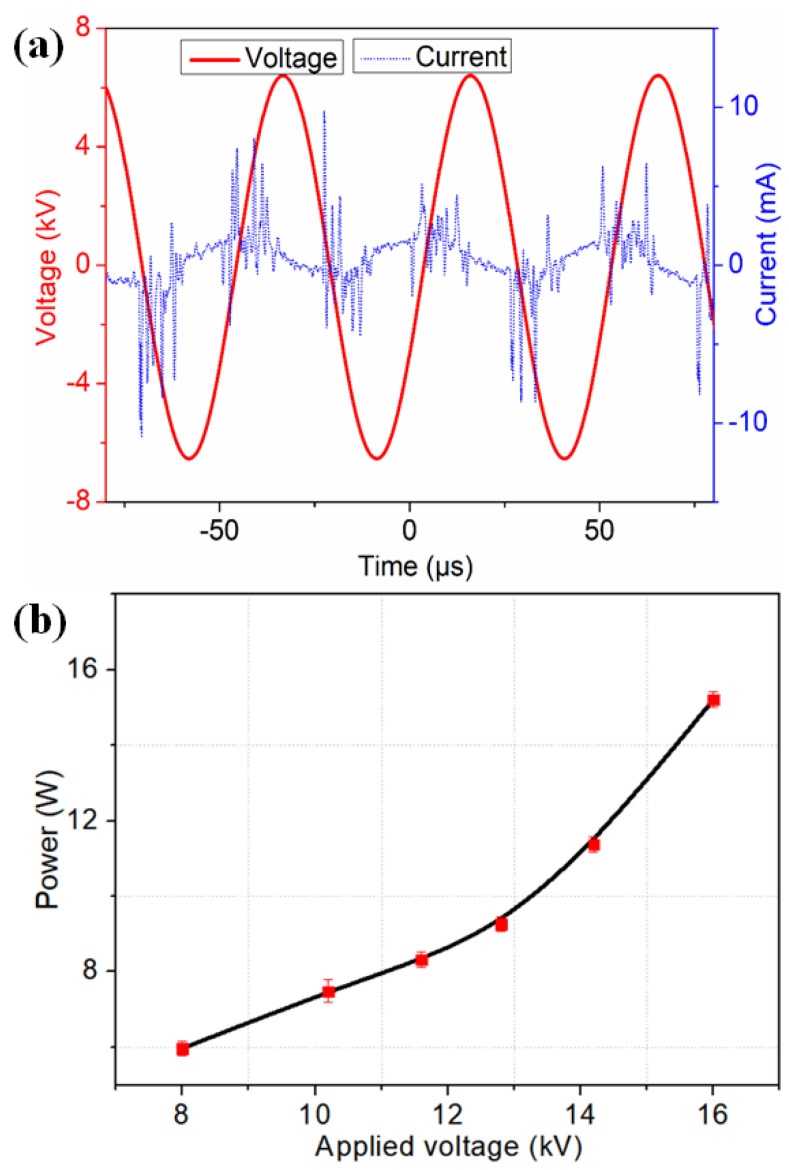
(**a**) Voltage–current discharge characteristics of Ar/H_2_O microplasma jet; (**b**) variation in dissipated power with different applied voltages.

The electrical characteristics of μAPPJ were demonstrated by the voltage–current (*V*–*I*) curves of the plasma discharge. A typical example of a *V*–*I* curve with an applied voltage of 12.8 kV (peak-to-peak voltage) at a frequency of 20 kHz and a water vapor content of 0.2% is shown in Figure 2a. It can be seen that, during both the positive and negative half-cycles of the applied voltage, multi-spikes with an amplitude of about 5–10 mA were observed in the current curve. These current spikes were considered as the filament discharge between the needle and the ring electrodes [22].

Dissipated power is another key parameter for the electrical characteristics of the μAPPJ, and its value was obtained by the following formula:

(1) $$P=\frac{1}{T}\int_{t}^{t+T}V(t)\times I\left(t\right)dt$$

where *P* represents the dissipated power, *T* is the period of the discharge and *V*(*t*) and *I*(*t*) are the voltage and current of the plasma discharge, respectively. Figure 2b shows the variation in the dissipated power with different applied voltages. It can be seen that the power increased from 5.9 to 15.2 W when the applied voltage (peak-to-peak voltage) varied from 8 to 16 kV.

### 3.2. OES of μAPPJ

**Figure 3:**
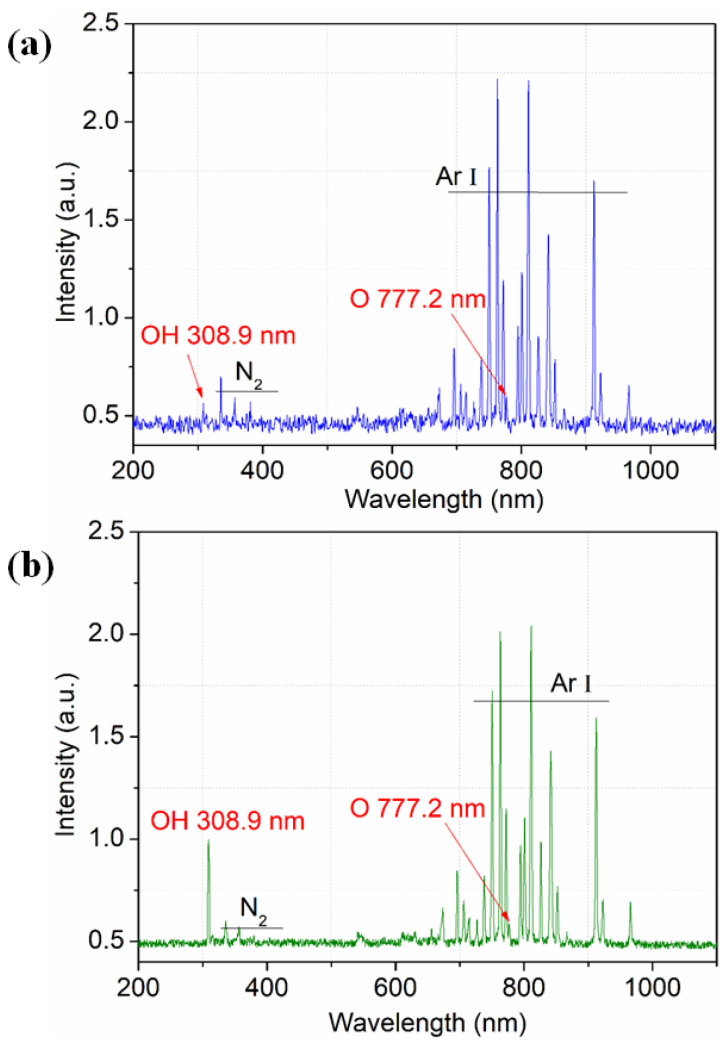
Emission spectra of microplasma jets excited by (**a**) pure Ar and (**b**) Ar/H_2_O in ambient air conditions.

To investigate the presence of reactive species generated in the plasma plume, the typical optical emission spectra of pure Ar and Ar/H_2_O microplasma jets are shown in Figure 3. Both the applied voltages were kept at 12.8 kV, and the water vapor content for the Ar/H_2_O microplasma jet was kept at 0.2%. It can be seen that both the spectra were dominated by neutral Ar atoms (Ar I) lying in the wavelength ranging from 700 to 850 nm. Due to the fact that the plasma was generated in open-air conditions, reactive O atoms at 777.2 nm, OH radicals at 308.9 nm and molecular nitrogen spectral bands in the range of 330 to 420 nm were also detected in both the pure Ar and Ar/H_2_O microplasma jets. These reactive species mainly came from the inelastic electron-impact collisions or the collisions between the Ar* and the O_2_ or water vapor in the ambient air, especially for the pure Ar microplasma jet [23]. When a certain amount of water vapor was introduced into the plasma, it could be clearly seen from the spectrum that the OH intensity increased in the Ar/H_2_O microplasma jet. The possible reaction pathways to generate the OH radicals are listed as Reactions (2)–(6). It can be concluded that the OH intensity is mainly dependent on the water vapor molecules and the electron density [24], and it plays an important role in polymer surface modification.

e + H_2_O ⇨ e + H^•^ + ^•^OH (2)

e + H_2_O ⇨ 2e + H^+^ + ^•^OH (3)

e + H_2_O ⇨ 2e + H_2_O^+^ (4)

e + H_2_O^+^ ⇨ e + H^+^ + ^•^OH (5)

Ar* + H_2_O ⇨ Ar + H^•^ + ^•^OH (6)

**Figure 4:**
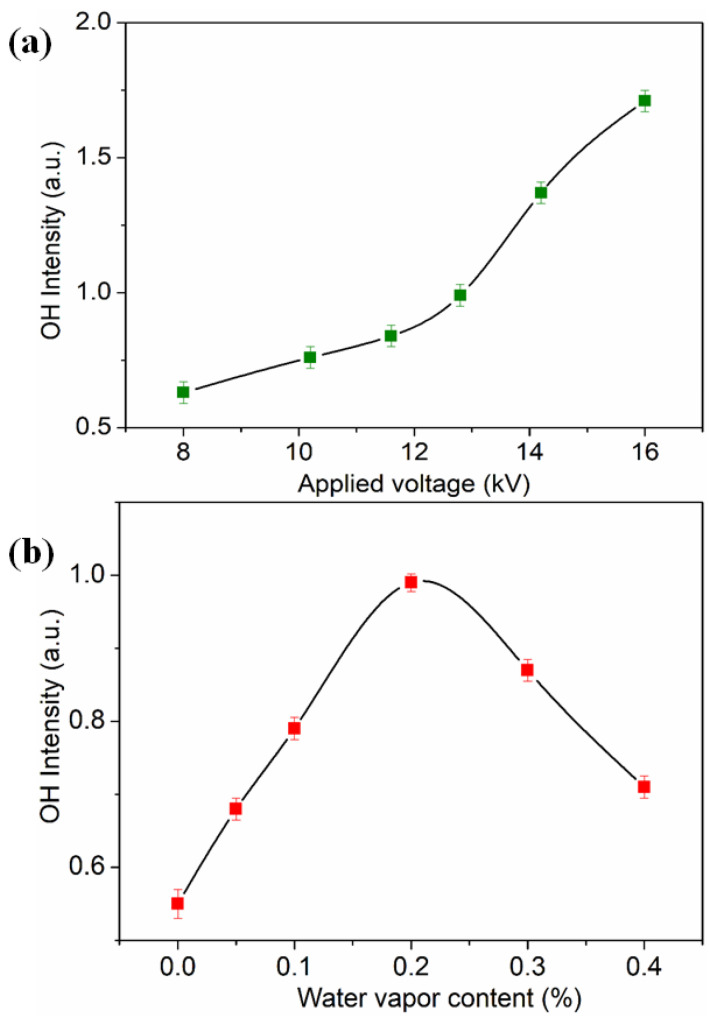
Variation in the intensity of OH spectra with different (**a**) applied voltages (water vapor content: 0.2%) and (**b**) water vapor content (applied voltage: 12.8 kV).

Figure 4a shows the emission intensity of the OH radicals as a function of the applied voltage. With an increase in the applied voltage, the intensity of OH increased due to the increase in the electron density, which provided more reactive particles for the surface modification of PI films. However, in the experiment, we found that the applied voltage should not be too high, especially for the microplasma jet generator with a tip nozzle of 100 microns. When the voltage was higher than 14.2 kV, its tip was easily damaged due to the discharge filaments. Therefore, it is necessary to control the applied voltage in the experiment to provide more reactive particles and ensure that the plasma jet generator is not damaged.

The effect of the water vapor content on the emission intensity of OH was also investigated, and the results are shown in Figure 4b. As the water vapor content increased from 0 to 0.4%, the intensity of OH at 308.9 nm first rapidly increased to the maximum value, and then gradually decreased with the further introduction of water vapor. The reason for this phenomenon is that water vapor is an electro-negative gas, and the electron density decreases with excessive water vapor content via electron attachment [25].

### 3.3. Hydrophilicity of PI Films Modified by μAPPJ

**Figure 5:**
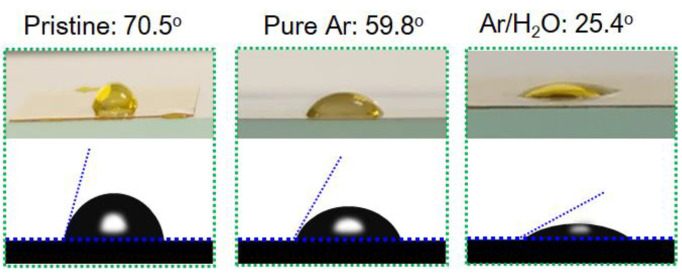
Water contact angle of untreated PI film and the PI films modified by pure Ar and Ar/H_2_O microplasma jets.

The hydrophilicity of the PI films was evaluated by water contact angle (WCA) measurements. Figure 5 presents the WCAs of an untreated PI film and the films modified by the pure Ar and Ar/H_2_O microplasma jets with an applied voltage of 12.8 kV and a water vapor content of 0.2%. The WCA of the pristine PI surface reached about 70.5°, and this value decreased to 59.8° after processing with a pure Ar microplasma jet for 60 s. When 0.2% water vapor was introduced into the plasma, the WCA of the PI surface further decreased to 25.4° with the same plasma treatment time. This confirms that the surface hydrophilicity of the PI film was greatly improved after the microplasma jet modification, and the addition of water vapor was beneficial for further improvement.

**Figure 6:**
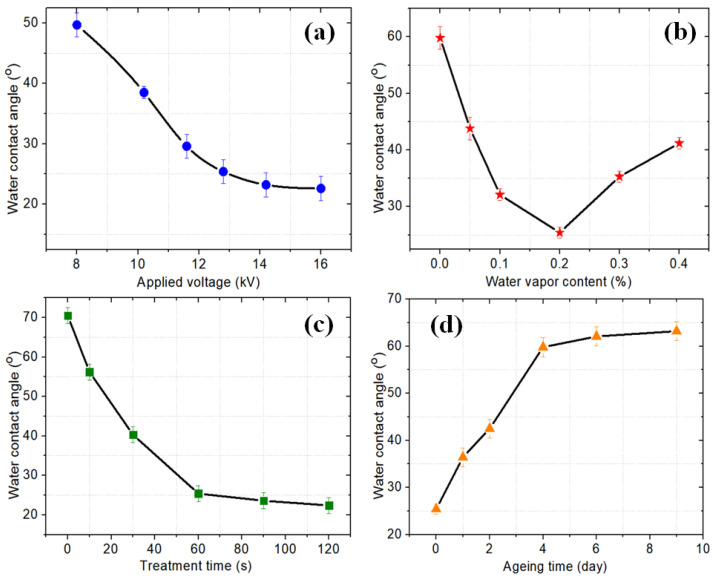
Water contact angles of plasma-modified PI films as a function of (**a**) applied voltage (water vapor content: 0.2%, plasma treatment time: 60 s), (**b**) water vapor content (applied voltage: 12.8 kV, plasma treatment time: 60 s), (**c**) plasma treatment time (applied voltage: 12.8 kV, water vapor content: 0.2%) and (**d**) aging time (applied voltage: 12.8 kV, water vapor content: 0.2%, plasma treatment time: 60 s).

Figure 6a–c show the variation in the WCAs of Ar/H_2_O microplasma-treated PI films with different applied voltages, water vapor contents and plasma treatment times, respectively. It can be seen that, with the other parameters unchanged, the WCA decreased greatly with increasing applied voltage. This result was mainly due to the fact that a higher voltage would produce more reactive particles (as shown in Figure 4a), such as OH, to participate in the surface modification reaction of the PI, resulting in a better hydrophilicity. However, we also found that when the voltage exceeded 16 kV, the WCA tended to saturate and no longer decreased. When the water vapor content increased from 0 to 0.2%, the WCA decreased rapidly, but with a further increase in the water vapor content, the WCA increased gradually, as shown in Figure 6b. It can be seen from the spectral results in Figure 4b that the OH intensity had a similar variation trend with the water vapor content. This result indicates that the OH concentration plays a key role in improving the hydrophilicity of PI surfaces. However, it is also necessary to control the water vapor content so that the modification effect can be optimized. Besides, the influence of the plasma processing time on the WCA of PI film surfaces was also investigated, where it can be seen that the WCA value decreased rapidly in the first 60 s and gradually decreased with longer plasma processing times.

Finally, the aging effect of the plasma-modified PI film was also investigated, as shown in Figure 6d. It can be seen that a major recovery of the WCA was observed in the first 4 days, while there was no significant increase after a storage time of 6 days with a WCA of 62.1°. This aging effect was mainly due to surface contamination and the orientation of polar groups [26]. In general, the WCA after aging was still smaller than that of the pristine PI film (70.5°).

### 3.4. AFM Morphologies of PI Films

**Figure 7:**
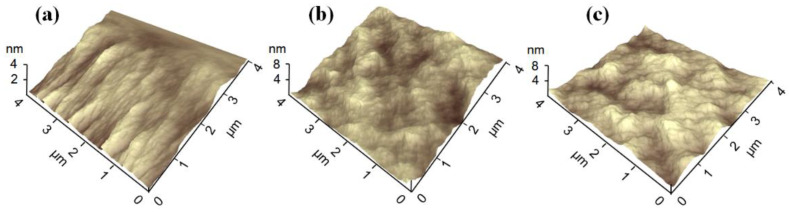
AFM images of (**a**) untreated PI film and plasma-modified PI films by (**b**) pure Ar and (**c**) Ar/H_2_O microplasma jets.

The physical and chemical interaction processes between the PI surface and the Ar/H_2_O microplasma jet were investigated in this work to further illustrate the hydrophilic variation in PI films modified by the μAPPJ. The micro-morphologies of the PI film surfaces were analyzed using AFM images. Figure 7 shows the AFM images of the pristine PI film and the plasma-modified PI films by pure Ar and Ar/H_2_O microplasma jets. It could be seen that the surface of the untreated PI film was generally smooth and flat, mainly with some stripe-like convex structures, and the average surface roughness was only 2.3 nm. After the μAPPJ treatment, the PI surfaces had lots of hill-like protrusions, and the film surface roughness also increased (about 3.4 to 4.1 nm). It is well known that plenty of high-energy particles, such as electrons, Ar^+^ and excited Ar, are generated in the plasma jet, and these particles will bombard and etch the PI film surface, resulting in undulating and rough surface morphologies [27]. A rougher surface of the PI films was beneficial for increasing the surface hydrophilicity [28].

### 3.5. XPS Analysis of PI Films

**Figure 8:**
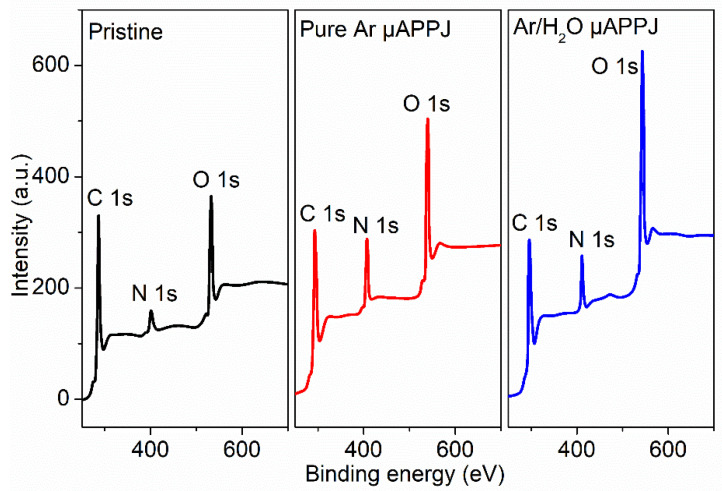
XPS spectra of pristine PI film and plasma-modified PI films by pure Ar and Ar/H_2_O microplasma jets.

**Figure 9:**
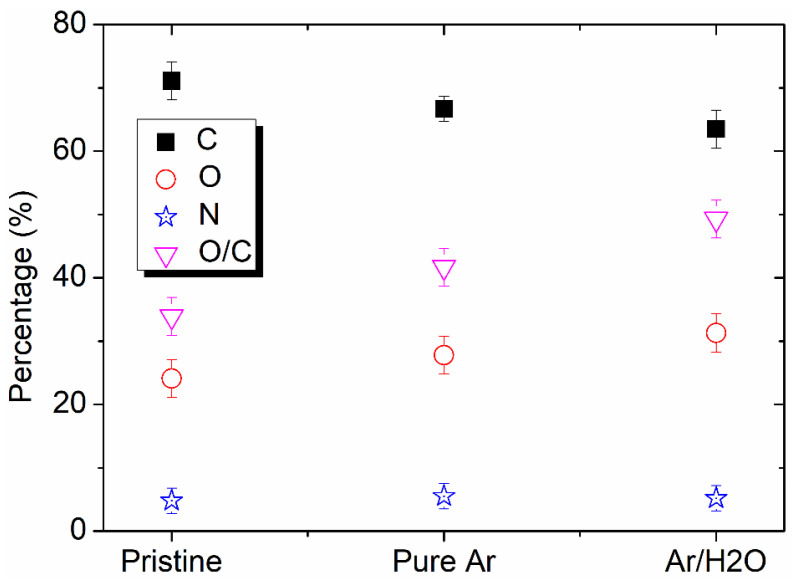
Relative percentages of C, O, N and O/C of pristine, pure Ar and Ar/H_2_O microplasma-jet-modified PI films.

**Figure 10:**
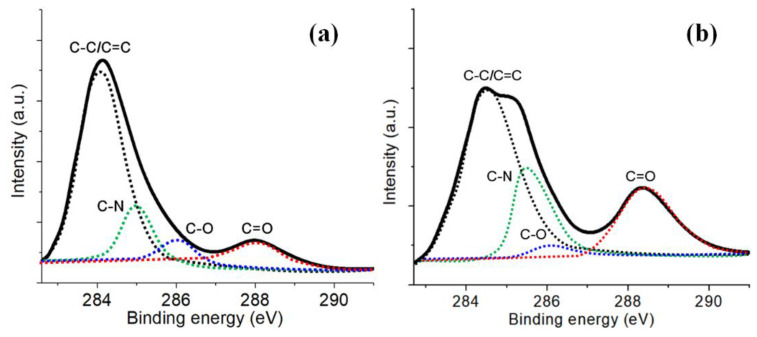
Deconvolution of C 1 s spectra of (**a**) pristine PI film and (**b**) PI film modified by Ar/H_2_O microplasma jet.

The chemical compositions of the PI surfaces before and after the plasma treatment were also examined to further clarify the hydrophilic effect of the plasma modification. Figure 8 presents the XPS spectra of the pristine PI film and the plasma-modified PI films by pure Ar and Ar/H_2_O microplasma jets. Compared with the pristine PI film, it was obvious that the intensity of the C 1 s peak decreased, while the intensities of the N 1 s and O 1 s peaks increased. Figure 9 shows the relative percentages of C, O, N and O/C in the pristine, pure Ar and Ar/H_2_O microplasma-jet-modified PI films. The increase in the O/C ratio on the plasma-treated PI surfaces indicated that the plasma induced surface functionalization of the PI surface, and the addition of water vapor was helpful in grafting more oxygen-containing functional groups. This inference was verified by the deconvolution of the C 1 s spectra of the untreated PI film and the PI films modified by the Ar/H_2_O microplasma jet, as shown in Figure 10. It can be seen that the C 1 s peak could be resolved into four main components: the peak at 284.2 eV represented the C-C aromatic carbon; the peak at 285.1 eV was assigned to C-N; the peak at 286.1 eV was denoted to C-O-C; and the peak at 288.2 eV belonged to the carboxyl groups, C=O-OH [22,28,29,30]. The increase in the concentration of C=O-OH on the PI surface after the Ar/H_2_O microplasma treatment was attributed to surface oxidation by oxygen-containing reactive species generated in the microplasma, such as OH. It is well known that these oxygen-containing functional groups are polar groups, which play a key role in improving the hydrophilic properties of the PI film surface [31,32]. This further confirmed that after the microplasma jet treatment, diverse hydrophilic oxygen-containing functional groups were grafted onto the surface of the PI film, which was the main reason why the surface of the PI film became hydrophilic. By adjusting the plasma parameters to change the content of these functional groups generated on the film surface, the hydrophilicity of the PI film surface can be adjusted [33].

### 3.6. Modification Mechanism of PI Film by Microplasma Jet

**Figure 11:**
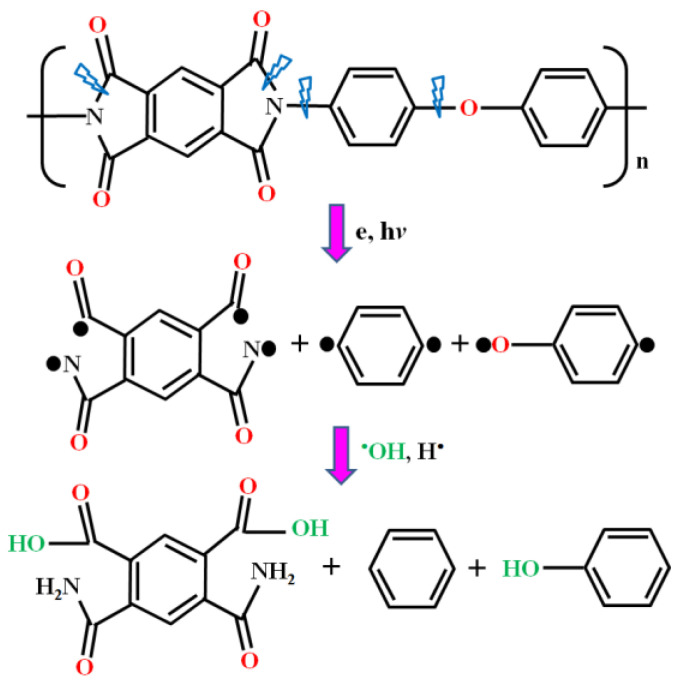
Modification mechanism of PI film by Ar/H_2_O microplasma jet in ambient air conditions.

The modification mechanism of a PI film surface by an Ar/H_2_O microplasma jet was discussed in this work through the interaction process between the PI surface and the plasma. From the results of the physical morphologies and chemical compositions of the PI films after plasma modification, it could be seen that the reactive and excited particles, free electrons contained in Ar/H_2_O microplasma, bombarded the molecular chains on the PI film surface, resulting in cross-linking and etching on its surface, and finally introducing diverse oxygen-containing reactive functional groups on the PI surface [34]. Figure 11 demonstrates the reaction processes on the PI surface during microplasma jet modification. Because the C-N bond and C-O bond in the PI molecular chains are weak, they will be broken first under the action of electrons and ultraviolet photons in the plasma [35]. These broken molecular chains then react with O atoms and OH radicals in the plasma to further form new functional groups, such as -NH_2_ and -C=O-OH.

## 4. Conclusions

In this paper, an atmospheric pressure Ar/H_2_O microplasma jet (μAPPJ) was used to achieve localized tailoring of the poor hydrophilicity of PI films. The electrical properties of the μAPPJ were detected and the results showed that plenty of discharge spikes occurred during the increasing and dropping slope of the voltage waveform, and that the dissipated power of the μAPPJ increased from 5.9 to 15.2 W when applied voltage varied from 8 to 16 kV. The OES results indicated that diverse reactive and excited species, such as nitrogen molecules, OH radicals, Ar* and O atoms, were successfully produced in the plasma plume. In addition, the emission intensity of OH increased when the applied voltage increased from 8 kV to 16 kV with a proper addition of water vapor (0.2%). As an electro-negative gas, an excessive water vapor content (>0.2%) would cause a reduction in the OH intensity. Compared with the WCA of a pristine PI surface (70.5°), the WCAs of plasma-modified PI surfaces were significantly reduced (25.4°), and the WCA value could be adjusted by choosing different plasma parameters, such as the applied voltage, water vapor content and plasma processing time. The aging process indicated that the WCA could be increased after 4 days of storage, but the hydrophilicity was still better than that of the pristine films. The AFM images revealed an increased surface roughness of the PI film after the microplasma modification. The XPS analysis proposed the generation of polar oxygen-containing functional groups, such C=O-OH and NH_2_, on the plasma-treated PI film surfaces. In addition, an increase in the roughness and the introduction of polar functional groups together led to an improvement in the wettability of the PI films. Finally, the processes of the destruction of C-N and C-O bonds and OH radical grafting were discussed, and they were considered to be the possible reaction mechanism between the PI film and the μAPPJ. This work could provide a reference for the localized surface hydrophilicity tailoring of polyimide films. In our future work, the maskless writing of hydrophilic micropatterns with different wettabilities will be explored with the aid of a three-axis mobile platform, and detailed applications of the modified PI films for wearable and implantable devices will be investigated.
